# Prediction of Concrete Compressive Strength in Saline Soil Environments

**DOI:** 10.3390/ma15134663

**Published:** 2022-07-02

**Authors:** Deqiang Yang, Changwang Yan, Shuguang Liu, Zhirong Jia, Chunguang Wang

**Affiliations:** 1 School of Civil and Architectural Engineering, Shandong University of Technology, Zibo 255000, China; chinaxq2015@163.com (D.Y.); cgwang@sdut.edu.cn (C.W.); 2 School of Mining and Technology, Inner Mongolia University of Technology, Hohhot 010051, China; ycw20031013@126.com (C.Y.); liusg6011@126.com (S.L.)

**Keywords:** saline soil, corroded concrete, time-dependent, compressive strength, corrosion effects

## Abstract

Saline soil in Western China contains high concentrations of chloride ions, sulfate ions, and other corrosive ions, and the performance of concrete will substantially deteriorate from exposure to this environment. Therefore, it is of great significance to study and predict the concrete compressive strength in saline soil environments. In this paper, the effects of corrosion on concrete were analyzed from the aspects of surface damage, damage depth, and X-ray diffraction (XRD) of the corrosion products. The effects of corrosion were quantified by damage depth and corrosion depth. Then, considering the corrosion effects combined with Fick’s diffusion law, a time-dependent model of concrete compressive strength and a time-dependent model of damage depth were established. The results show that the deterioration of concrete gradually developed from the surface to the interior, and that the interface of the concrete specimen was equivalent to three parts: a failure zone, a filling zone, and an undisturbed zone. The results also showed that the time-varying model of concrete compressive strength proposed by the author was fully applicable, with an error of less than five percent. The service life of concrete predicted by the damage depth was found to be about 253 months (21.1 years), and the service life predicted by the time-varying compressive strength model was about 187 months (15.6 years). Both prediction results were far less than the normal concrete service life of 50 years. In addition, the long-term compressive strength of the corroded concrete was about 90% of that of the noncorroded concrete, which did not deteriorate with the corrosion time.

## 1. Introduction

The compressive strength of concrete is substantially deteriorated by exposure to a corrosive environment and this deterioration greatly reduces the service life of a concrete structure [1,2]. Therefore, accurately evaluating concrete compressive strength is highly important both for research and for engineering applications [3]. There are many types of corrosive media that can affect the compressive strength of concrete, among which chloride [4,5] and sulfate [6,7] are the most notable. When chloride salt enters concrete, it mainly reacts with the Tricalcium aluminate (C_3_A) [8] in concrete and produces Friedel’s salt (C_3_A·CaCl_2_·10H_2_O) [9,10], which does not have cementitious properties; this phenomenon causes the concrete to deteriorate and the compressive strength to reduce. Sulfate physically and chemically attacks concrete [11]. The physical attack from sulfate mainly causes the expansion and destruction of the internal structure of concrete through the crystal expansion of Na_2_SO_4_·10H_2_O [12,13]. The chemical attack from sulfate is due to the chemical reaction. Normally, alumina-bearing phases and calcium hydroxide are more vulnerable to sulfate attacks than other compounds present in hydrated Portland cement paste. The secondary ettringite may be formed from components of the monosulfate phase, Ca_4_Al_2_O_26_H_38_, Ca_3_Al_2_O_6_, and Ca_4_Al_2_Fe_2_O_10_, among other alumina-bearing phases present in hydrated cement-based materials [14]. The gypsum crystal (CaSO_4_·2H_2_O) [15] and ettringite crystal (3CaO·A1_2_O_3_·3CaSO_4_·31H_2_O) [16] are two type of expansion crystals, which grow in the original pores of concrete to increase compactness and compressive strength [17]. However, these crystals can also cause large expansion stresses on the inner walls of the pores in concrete [18,19], causing damage and microcracks which deteriorate the concrete and reduce the compressive strength.

In view of the mechanism of concrete degradation in the abovementioned chloride and sulfate environments, many scholars have studied the compressive strength of concrete which has been exposed to corrosive environments. Some scholars have found that the compressive strength tends to gradually decrease after exposure to corrosive environments for different durations [20,21], and some scholars believe that the compressive strength first increases and then decreases [22,23,24]. In recent years, many predictive models have been established for the corroded concrete compressive strength, among which the corrosion coefficient [25,26] has been used to analyze the relationship between the compressive strengths of corroded and noncorroded concrete. However, these models do not distinguish the different properties between the corroded zone and the noncorroded zone of concrete specimens, i.e., these models were established by using the corroded concrete specimens as a whole. Furthermore, the corroded concrete specimens are divided into two stages—the strengthening stage and the deterioration stage—according to the exposure time [27,28]. This model divides the effect of sulfate on concrete according to the exposure time into an early strengthening effect and a later deteriorating effect. In fact, the two effects are simultaneous inside the concrete and will not be different because of different exposure times. Therefore, in this paper, two kinds of corrosion effects of sulfate on concrete are defined by damage depth and corrosion depth. The corroded concrete specimens with arbitrary exposure times are divided into three parts: a failure zone, a filling zone, and an undisturbed zone. Different exposure times create variations in the damage depth and corrosion depth, which lead to continuous changes in the areas of these three zones and in the compressive strength of the concrete.

Saline soils, which contain high concentrations of Cl^−^, SO_4_^2−^, CO_3_^2−^, Na^+^, K^+^, and Mg^2+^, are widely distributed in Northwest China [29,30]; these corrosive ions have played a significant role in the deterioration of concrete [31,32,33]. Moreover, the diffusion rate of ions in the local dry and wet environments is accelerated [34]. In this paper, first, the test solution was allocated according to the composition of saline soil, and then six groups of concrete specimens were tested for dry and wet cyclic corrosion and compression strength at different exposure times. Second, the effects of corrosion on concrete were analyzed from the aspects of surface damage and damage depth, and from the X-ray diffraction (XRD) results of the corrosion products. Third, the effects of corrosion were quantified by damage depth and corrosion depth, wherein the interface of the concrete specimen was equivalent to three parts: a failure zone, a filling zone, and an undisturbed zone. Fourth, combined with Fick’s diffusion law, a mechanical model of concrete compressive strength was established, which considers the abovementioned corrosion effect. Finally, the predicted model of concrete service life was obtained by fitting the chloride surface content and the diffusion coefficient.

## 2. Materials and Methods

### 2.1. Materials

The test piece was a cubic specimen with side lengths of 100 mm. The total number of test pieces was 36. The chemical composition and the working performance index of the cement P. O. 42.5 R, according to Chinese Portland cement Standard [35], similar to ASTM Type I ordinary Portland cement, are shown in Table 1 and Table 2, respectively. The fineness modulus of the natural river sand used was 2.8, and its apparent density was 2600 kg/m^3^. The size of the crushed stone was 5~25 mm, and its apparent density was 2660 kg/m^3^. The mix of concrete is shown in Table 3.

The manufacturing process of the test piece is as follows: (1). Weigh the cement, stone, sand, and water used in the test according to mass mix of concrete in Table 3. (2). Add the stones, sands, and cement into the cement mixer in turn and start it, mixing the dry materials evenly. Then, add a small amount of water several times until the concrete is mixed evenly. (3). Put the mixed concrete into the plastic mold coated with release agent (the internal dimension of the mold was 100 × 100 × 100), then place the mold on the cement vibrating table for vibration and smooth the concrete surface. (4). Cover the concrete surface with plastic film and place it at room temperature for 24 h, then demold the concrete surface and put the prepared concrete specimen into the concrete standard curing box (temperature 23 °C, relative humidity 95%) to cure for 28 days.

### 2.2. Test Methods

#### 2.2.1. Dry and Wet Cycle Corrosion Test

There were 6 dry and wet cycle inspection periods in total: 0 months (not corroded, as a comparative test), 5 months, 8 months, 10 months, 15 months, and 20 months. Six cubic specimens were soaked in each inspection period, with a total number of 36 specimens. The test solution was composed of the same composition and mixture ratio as saline soil in Western China [36]. The mix of the test solution is shown in Table 4, and the ion concentration in saline soil and test solution is shown in Table 5. A complete dry and wet cycle inspection period included soaking in solution for 15 days and natural air drying for 15 days.

#### 2.2.2. Determination of Ion Content and Diffusion Depth Test

After the dry and wet cyclic corrosion test of each group of concrete specimens was completed, compressive tests were carried out on three of the concrete specimens. Subsequent analyses of the free chloride content and the free sulfate content and the XRD analysis of the corrosion products were carried out on the other three specimens.

When testing the distribution of ions in concrete, the corresponding test specimens were ground and sampled. The sampling depth was within 20 mm from the concrete surface, and samples were taken every 2 mm. After exceeding 20 mm, samples were taken every 5 mm—that is, the sampling depths were 1, 3, 5, 7, 9, 11, 13, 15, 17, 19, 22.5, 27.5, and 32.5 mm, respectively. After the sample was taken, it was then passed through a 0.63 mm sieve to remove large particles. According to the relevant Chinese standard [37], the free chloride content was determined by the Mohr method, and the free sulfate content was determined by ultraviolet spectrophotometer method.

##### Determination of Free Chloride

1. Prepare K_2_CrO_4_ indicator with a concentration of about 5%, phenolphthalein solution with a concentration of about 0.5%, dilute sulfuric acid, and standard NaCl solution with a concentration of 0.02 mol/L. Prepare a V_2_ mL standard silver nitrate solution with a concentration of about 0.02 mol/L with a V_1 mL_ standard NaCl solution. The calculation formula of its concentration *C*_AgNO3_ is shown in (1),
  (1) $$C_{{\mathrm{AgNO}}_{3}}=C_{\mathrm{NaCl}}\times \frac{V_{1}}{V_{2}}$$
2. Place the concrete sample in the drying box oven for 2 h with a drying temperature of 105 ± 1 ℃ to ensure that the water in the sample is fully volatilized. Accurately weigh 2 g (recorded as G) of the dried sample and pour it into a triangular beaker. Add 200 mL (V_3_) of distilled water, tighten the cork, and shake it violently for 1–2 min. Soak it for 24 h. Shake it again after soaking for 12 h to ensure that the chloride ions in the sample are fully dissolved in the distilled water.
3. Use neutral medium-speed filter paper to filter out the sediment in the soaking solution; measure two 20 mL (V_4_) portions of filtrate, respectively, and put them into two triangular flasks; add two drops of phenolphthalein to the two triangular flasks, respectively, and then neutralize them with dilute sulfuric acid until they are colorless to ensure that the filtrate is neutral. Add 10 drops of potassium chromate indicator into the triangular flasks, and then titrate them with silver nitrate solution immediately until brick red sediment appears. Record the volume of silver nitrate solution (V_5_) consumed (the triangular flask needs to be shaken violently during titration). The calculation formula of free chloride ions is:
  (2) $$c_{f}=\frac{C_{{\mathrm{AgNO}}_{3}}\times V_{5}\times 0.03545}{G\times \frac{V4}{V_{3}}}\times 100$$
  where *c_f_* is the content of free chloride ions in the sample (%, indicating the percentage of chloride ions in the concrete mass); *C*_AgNO3_ is the concentration of silver nitrate solution (mol/L); G is sample mass (g); V_3_ is the volume of distilled water added to the sample (mL); V_4_ is the volume of filtrate used for each titration (mL); and V_5_ is the volume of silver nitrate solution consumed after titration (mL). The final measured value of *c_f_* is the average value of the two measured results.

##### Determination of Free Sulfate

**Figure 1:**
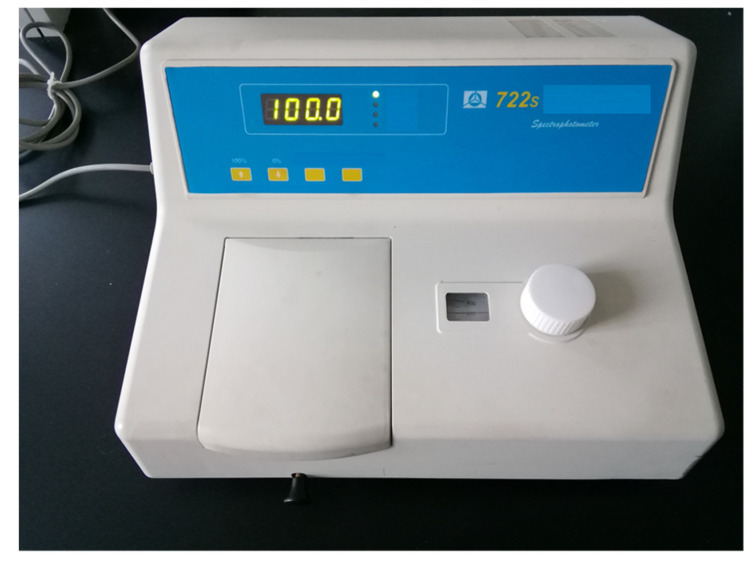
Ultraviolet spectrophotometer.

Ultraviolet spectrophotometer method was used to measure the content of free sulfate ions, as shown in Figure 1. BaCl_2_-PVA (polyvinyl alcohol) mixture was prepared in advance, and then 2.0 g of concrete powder sample was weighed, soaked in 50 mL distilled water, shaken for 2 h, and then soaked for more than 24 h. The sample was filtered with slow-speed filter paper. An amount of 25 mL filtrate was taken into a 50 mL volumetric flask. First, 2.5 mL hydrochloric acid was added to the volumetric flask, followed by 10 mL of uniformly mixed BaCl_2_-PVA mixture. The volume of distilled water was fixed to 50 mL, and the turbidity of the solution was shaken 2–3 times by hand. After the turbidity of the solution was obviously stable, the solution was left to stand for 5 min and then tested by ultraviolet spectrophotometer. The relationship between the SO_4_^2-^ content measured in the test and the absorbance value is as follows:

*c_fs_* = 0.13281 + 1.42524 × abs + 11.7745 × abs^2^       *R*^2^ = 0.99209 (3)

where *c_fs_* is the mass of sulfate ions in the 50 mL solution (mg) and abs is the absorbance value displayed on the spectrophotometer display.

According to the absorbance value abs measured by the spectrophotometer, the free sulfate ion concentration can be calculated.

##### Determination of the Chloride Diffusion Depth

Assuming that all ions had the same damage depth in concrete during the same dry and wet cyclic corrosion duration, the damage depth was expressed by the chloride diffusion depth in this paper, and the chloride diffusion depth was determined by the AgNO_3_ coloration method [38].

First, the corresponding specimen was cut from the middle part, and two planed sections were selected as the test surface. Then, 0.1 mol/L silver nitrate solution was sprayed evenly on the concrete section. In the area where the chloride ion concentration was greater than a certain critical value, the chloride ions reacted with the silver ions to form a white AgCl precipitation. When the chloride ion concentration was lower than a certain value, the hydroxyl ions reacted with the silver ions to form AgOH, and then oxidized to a brown Ag_2_O precipitation. An obvious color boundary, namely a color trace, formed at the junction of the two different color regions. Finally, vernier calipers were used to measure the depth of the color-developing area of the specimen section. Usually, the average width from the penetration surface to the color-changing boundary was measured as the average erosion depth of chloride ions.

#### 2.2.3. SEM-EDS Test

After the dry–wet cycle erosion test was completed and the corresponding specimens were taken for microscopic observation and corrosion product analysis. First, the specimens were cut and sampled at the depth of 10~15 mm from the concrete surface. After the test sampling was completed, the coarse aggregate in the samples was removed with a 200 mesh sieve, and then the carbon spraying treatment was carried out. Then, the SEM-EDS test was carried out on a SIGMA 500 field emission scanning electron microscope manufactured by Zeiss AG of Jena, Germany. The magnification of the concrete SEM image was 2000 times. Finally, the change of the internal microstructure of the concrete specimen was analyzed through the test results. EDS analysis was carried out by X pert3 powder X-ray diffractometer to detect the types of elements contained in the sample and to study the corrosion products generated in the concrete after erosion. XRD test conditions: Target was Cu Target, acceleration voltage was 40 kV, current was 40 mA, and scanning angle was 5–80°.

#### 2.2.4. Compression Strength Test

The electro-hydraulic servo universal testing machine, with a maximum range of 1000 kN, was used as the basic mechanical property test instrument of concrete. According to the relevant requirements in the national standards of China [39], the cube compressive strength was tested by a load-controlled loading system, at a loading speed of 0.5 MPa/s. The compressive strength value was taken as the average value measured by 3 test specimens.

## 3. Test Results and Analysis

### 3.1. Failure Patterns and Compressive Strength

**Figure 2:**
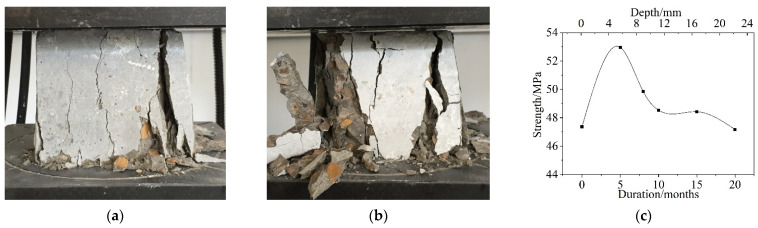
Failure patterns and variation in compressive strength after different dry and wet cycle inspection periods. (**a**) 5 months, (**b**) 10 months, (**c**) Variation in compressive strength.

The failure patterns and variations in the compressive strength after different dry and wet cycle inspection periods is shown in Figure 2. The failure characteristics of the corroded concrete cube specimens under uniaxial compression were essentially the same as those of uncorroded concrete. There were no visible cracks on the surface of the specimens before the load reached the peak values. However, when the load reached the peak values, a number of discontinuous longitudinal cracks formed on the surface of the specimen, parallel to the direction of the applied load. Keeping the load unchanged, the longitudinal cracks developed rapidly and penetrated the entire specimen, as shown in Figure 2a. When the concrete specimens were substantially corroded, the concrete peeling was severe during the failure of the test specimen, and there was a local crushing phenomenon, as shown in Figure 2b.

According to the relevant Chinese code [39], the compressive strength of a cube is calculated with the following formula:

(4) $$f_{cu}=0.95\cdot \frac{F}{A}$$

where *f_cu_* is the compression strength (MPa), 0.95 is the size conversion factor, *F* is the failure load (N), and *A* is the area of the cross section (mm^2^).

As can be seen from Figure 2c, the compressive strength increased rapidly from 0 to 5 months. At 5 months, it reached a maximum value, which was approximately 10~12% higher than that of the noncorroded concrete specimens. The compressive strength decreased rapidly from 5 to 10 months. After 10 months, the compressive strength decreased gradually. At 20 months, the compressive strength was slightly less than that of the noncorroded specimens. This finding shows that, as a result of the increase in the wet–dry cyclic corrosion duration, the compressive strength of concrete in the saline soil environment first increased and then decreased. Hence, the composition and microstructure of concrete underwent a change from compactness to looseness after being corroded by saline soil, which caused a change in the mechanical properties of the concrete materials at different depths.

### 3.2. Surface Damage

**Figure 3:**
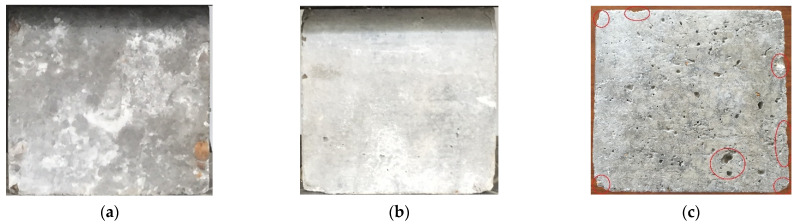
Surface changes in concrete after different dry and wet cycle inspection periods. (**a**) 5 months, (**b**) 15 months, (**c**) Surface pits and damage.

The surface changes in concrete after different dry and wet cycle inspection periods are shown in Figure 3. There was no obvious change in the surface of the specimens after 5 months of corrosion; white crystalline substances appeared in some areas, as shown in Figure 3a, which is referred to as the “salting out” phenomenon. When the specimen was in the natural air-drying state, the water in the concrete evaporated, and when the concentration of salt reached the supersaturated state, it precipitated on the surface of the concrete in crystalline form; thus, the “salting out” phenomenon appeared, and this is a physical change process. As the dry and wet cyclic corrosion duration increased, the concentration of the salt solution in the concrete became higher, and the “salting out” phenomenon became more substantial. Finally, the white crystalline materials covered approximately the whole surface of the specimen, as shown in Figure 3b. The surface morphology of the concrete specimens after removing the white crystalline material is shown in Figure 3c. The surface of the concrete became obviously rough, the number of holes increased, and some edges and corners of the concrete specimens were missing, the red circle part refers to the holes and peeled cementitious materials on the concrete surface. The reason for these changes is because the corrosion made the surface of the concrete exhibit a “sanding” phenomenon, which shows that the cement was dissolved, thereby exposing exposed sand and gravel.

**Figure 4:**
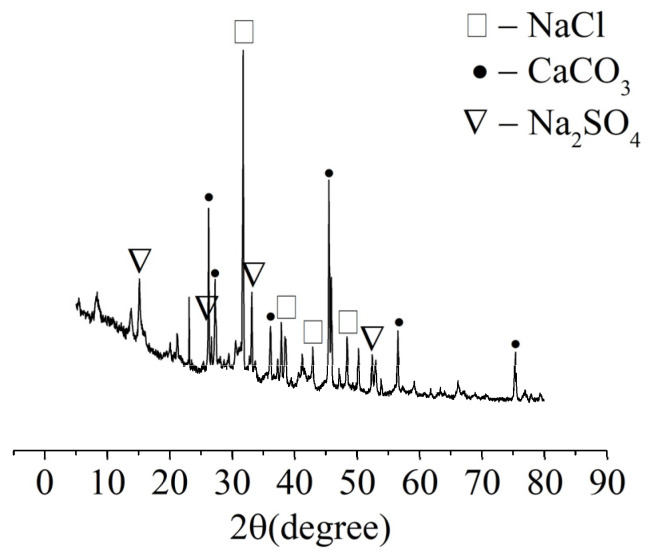
XRD analysis of the crystalline material on the surface of the specimens.

The XRD analysis was carried out on samples of the white crystalline substances from the “salting out” phenomenon on the concrete surface, and the results are shown in Figure 4. The main components of the white crystalline material were NaCl, Na_2_SO_4_, and CaCO_3_. The test solution contained NaCl and Na_2_SO_4_, but did not contain Ca^2+^, indicating that the Ca^2+^ came from the concrete. The test solution reacted with Ca(OH)_2_ and C-S-H in the concrete to form soluble calcium-containing substances, which precipitated from concrete in the form of CaCO_3_. The contents of Ca(OH)_2_ and C-S-H gel in the concrete decreased due to the precipitation of calcium ions, resulting in decreased concrete strength.

### 3.3. Internal Damage

#### 3.3.1. Damage Depth

**Figure 5:**
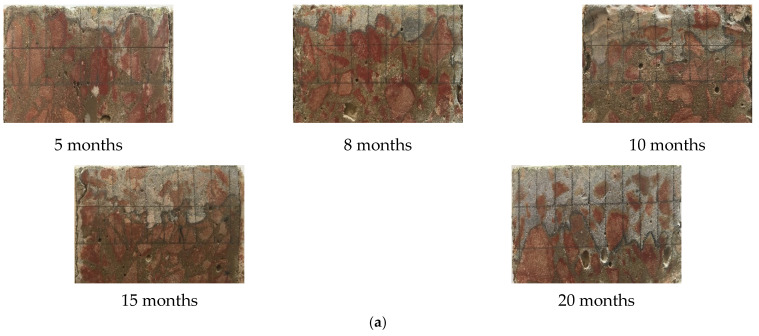

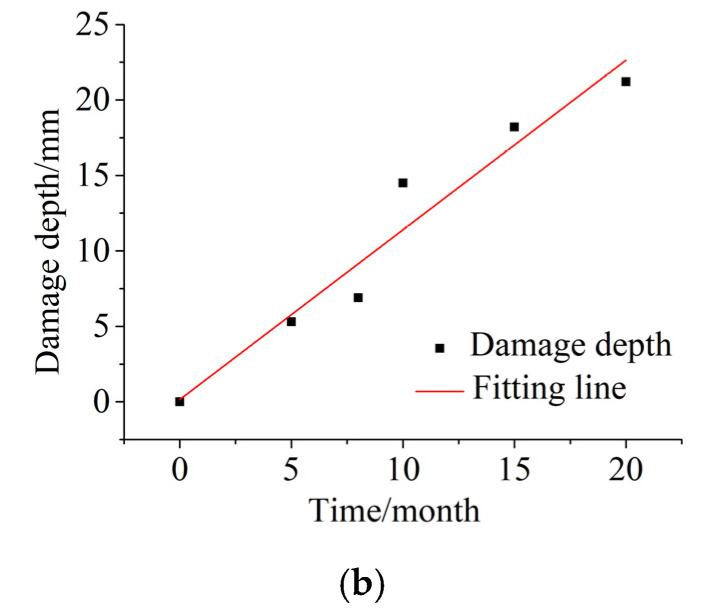
Damage depths of the specimens after different dry and wet cycle inspection periods. (**a**) AgNO_3_ coloration method for representing the damage depth of specimens after different dry and wet cycle inspection periods. (**b**) Variation in the measured damage depth after different dry and wet cycle inspection periods.

The damage depths of the specimens after different dry and wet cycle inspection periods are shown in Figure 5. In Figure 5a, the white area is AgCl precipitation, the red area is coarse aggregate, and the red–white color boundary is the damage depth. As the corrosion duration increased, the white area became larger and larger, and the color boundary became deeper. This is because the C_3_A only fixes chloride ions after an initial period, which is longer than the one corresponding to the absorption process. In contrast, the effect of the C_4_AF fixing chlorides by forming chloroaluminates is significant [40]. This finding shows that, as the corrosion duration increased, chloride gradually entered the interior of the concrete, and deterioration gradually developed from the surface to the interior of the concrete; Ma [29] reached the same conclusion. Moreover, this finding is consistent with Figure 5b. The damage depth of chloride increased linearly with increasing corrosion duration.

#### 3.3.2. Corrosion Products

**Figure 6:**
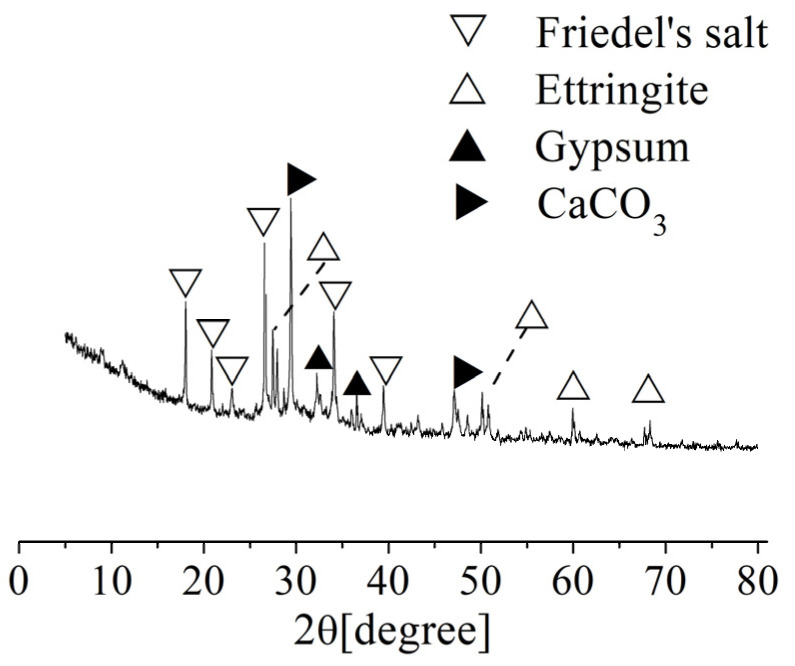
XRD analysis of the corrosion products of the concrete after corrosion for 10 months.

The corrosion products of the concrete specimens after corrosion for 10 months were analyzed by XRD at the distance of 10~15 mm from the surface of the concrete, as shown in Figure 6. The results show that the main corrosion products included ettringite crystal (3CaO·Al_2_O_3_·3CaSO_4_·32H_2_O), gypsum crystal (CaSO_4_·2H_2_O), Friedel’s salt (3CaO·AI_2_O_3_·CaCl_2_·10H_2_O), CaCO_3_ crystal, and other substances. The frontal angles of ettringite crystal are mainly 27°, 50°, and 60°. The frontal angles of gypsum crystal are mainly 32° and 36°. The frontal angles of Friedel’s salt are mainly 18°, 21°, 26°, 34°, and 39°. The frontal angles of CaCO_3_ crystal are mainly 29° and 47°. Both gypsum [15] and ettringite [16] have typical volume expansion, which have two effects on the internal structure of concrete. On one hand, these two crystals will grow in the original pores of the concrete to increase the compactness and compressive strength [17]. On the other hand, when the original pores of the concrete are fully filled by those two crystals, the continuous formation of crystals will produce internal pressure on the inner walls of the pores. When the internal pressure is greater than the tensile limit of the concrete, microcracks will form, thereby deteriorating the concrete and reducing the strength of the concrete. Moreover, Friedel’s salt, which is produced by the reaction of Cl^-^ and the cement hydration product (C–S–H gel), has no cementitious properties; therefore, the formation of Friedel’s salt results in a decrease in the cementing ability of the concrete. The above analysis shows that the corrosion effect is the main reason that the compressive strength of concrete first increases and then decreases.

## 4. Compressive Strength Mechanical Model Considering the Corrosion Effects

### 4.1. Mechanical Model of Compressive Strength

**Figure 7:**
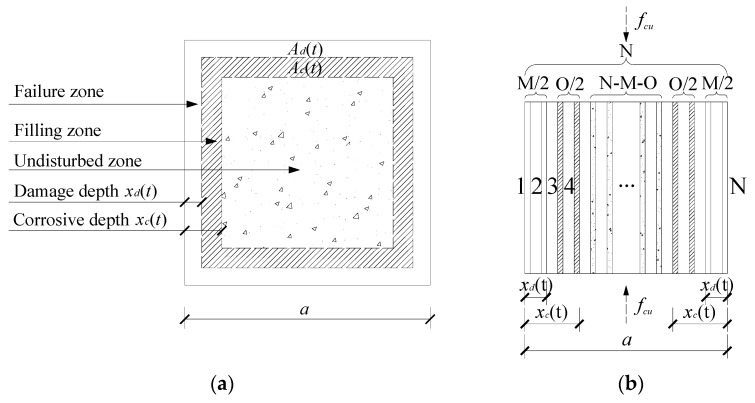
Mechanical model of compressive strength considering the corrosion effects. (**a**) Corrosion effects, (**b**) Parallel bar system.

As mentioned above, whether the corrosion effects fill or expand the internal pores of concrete depends mainly on the relationship between the content of the corrosion products and the internal porosity of the concrete [41]. The distribution of the corrosive ion content in concrete conforms to Fick’s diffusion law [42]; thus, the content of corrosive products decreases gradually with increasing concrete depth. The cross section of the concrete specimens was equivalent to three parts: a failure zone, a filling zone and an undisturbed zone, from outside to inside, by damage depth *x_d_*(*t*) and corrosion depth *x_c_*(*t*), as shown in Figure 7a. In the failure zone, the content of corrosive products was high, which mainly destroyed the internal structure of the concrete. In the filling zone, the content of corrosive products was low, which mainly filled the internal structure of the concrete. In the undisturbed zone, ions failed to reach this area.

It is assumed that the concrete is a homogeneous material. When the concrete is compressed, the concrete can be divided into N vertical rods. It was also assumed that the properties of each rod were the same, that the cross-sectional area was S, and that the failure stress was σ. Then, we were able to quantify the corrosion effect by the difference in the property and number of rods, as shown in Figure 7b. The initial cross-sectional area *A* and compressive strength of the concrete can be expressed as:

(5) A=N⋅S

(6) $$f_{cu}\left(0\right)=N\cdot \sigma$$

In the failure zone, there were M bars failure. The stress that each rod can bear is $\sigma_{d}$, where $\sigma_{d}=\gamma_{d}\cdot \sigma$. $\gamma_{d}$ is the corrosion damage coefficient ($\gamma_{d}\in \left[0,1\right]$). The cross-sectional area *A_d_* of the failure zone can be expressed as:

(7) $$A_{d}=M\cdot S$$

In the filling zone, there were O bars strengthened. The stress that each rod can bear is $\sigma_{c}$, where $\sigma_{c}=\gamma_{c}\cdot \sigma$. $\gamma_{c}$ is the corrosion enhancement coefficient. The cross-sectional area *A_c_* of filling zone can be expressed as:

(8) $$A_{c}=O\cdot S$$

The parallel bar model of compressive strength of corroded concrete considering corrosion effect can be expressed as:

(9) $$f_{cu}\left(t\right)=\left(N-M-O\right)\cdot \sigma +M\cdot \sigma_{d}+O\cdot \sigma_{c}$$

In conjunction with the above formulas, Formula (10) can be obtained:

(10) $$\begin{array}{l} f_{cu}\left(t\right)=\left(\frac{N-M-O}{N}+\gamma_{d}\cdot \frac{M}{N}+\gamma_{c}\cdot \frac{O}{N}\right)\cdot f_{cu}\left(0\right)=\left[1-\left(1-\gamma_{d}\right)\cdot \frac{A_{d}}{A}-\left(1-\gamma_{c}\right)\cdot \frac{A_{c}}{A}\right]\cdot f_{cu}\left(0\right)\\ =\left[1-\left(1-\gamma_{d}\right)\cdot \frac{a^{2}-{\left(a-2x_{d}\left(t\right)\right)}^{2}}{a^{2}}-\left(1-\gamma_{c}\right)\cdot \frac{{\left(a-2x_{d}\left(t\right)\right)}^{2}-{\left(a-2x_{c}\left(t\right)\right)}^{2}}{a^{2}}\right]\cdot f_{cu}\left(0\right) \end{array}$$

where *f_cu_*(*t*) is the compressive strength of concrete after corrosion for *t* month; *f_cu_*(0) is the compressive strength of noncorroded concrete; *A_d_*(*t*) and *A_c_*(*t*) are the cross-sectional areas of the failure zone and filling zone, respectively; *A* is the total cross-sectional area of the cross section; and *a* is the length of cross section.

### 4.2. Determination of Model Parameters

#### 4.2.1. Corrosion Damage Coefficient $\gamma_{d}$ and Corrosion Enhancement Coefficient $\gamma_{c}$

Various concrete specimens buried at Dun-huang Station in the saline soil area of Western China in 1959 were excavated and measured in 1995 after being buried in the soil for 36 years. After the corrosion of saline soil, the compressive strength of the ordinary Portland cement concrete tested in 1995 was approximately 10% lower than the original strength [43]. Kwon [44] placed ordinary concrete specimens on the Offshore Platform Marine Electrochemistry Center in India for a 10 year exposure test, resulting in a reduction in the concrete compressive strength of approximately 10% in both the immersion zone and in the splash zone. Therefore, the value of the corrosion damage coefficient *γ_d_* in this paper was 0.9.

Kumar and Bhattacharjee [45] established a relationship between the porosity, the average distribution pore size, and the compressive strength as:

(11) $$f_{c}\left(t\right)=1749\cdot \omega \cdot \left(\frac{1-p\left(t\right)}{\sqrt{r_{m}}}\right)$$

where *f_c_*(*t*) is the compressive strength when the porosity is *p*(*t*), *ω* is the cement content of the concrete, and *r_m_* is the average pore size distribution. The average *r_m_* of the concrete specimens after different dry and wet cyclic corrosion durations measured in this paper was 20.4 nm.

**Figure 8:**
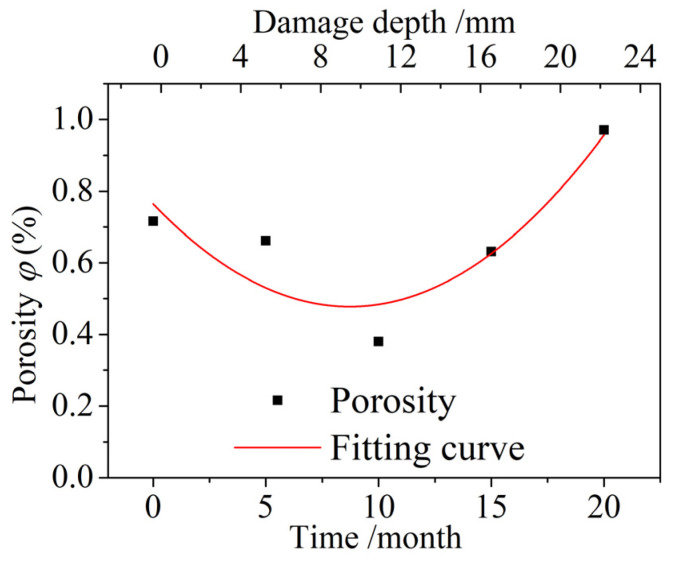
Variation in the porosity after different dry and wet cycle inspection periods.

The porosities of the concrete specimens taken 7–12 mm away from the surface of the concrete were measured, and the variation in the porosities after different dry and wet cycle inspection periods is shown in Figure 8. The porosity first decreased and then increased with increasing dry and wet cyclic corrosion durations, and the porosity was smallest after corrosion for 10 months. This finding shows that the concrete underwent a process of compaction before loosening. The relationship between the porosity *p*(*t*) of the concrete specimens and the dry and wet cyclic corrosion duration *t* is as follows:

(12) $$p(t)=0.0038t^{2}-0.0657t+0.7646$$

The corrosion enhancement coefficient *γ_c_* can be expressed by Formula (10):

(13) $$\gamma_{c}=\frac{f_{c}\left(t\right)}{f_{cu}(0)}=\frac{1749\cdot \omega \cdot \left(\frac{1-p\left(t\right)}{\sqrt{r_{m}}}\right)}{f_{cu}(0)}\approx 1.35$$

#### 4.2.2. Damage Depth *x_d_*(*t*) and Corrosion Depth *x_c_*(*t*)

The diffusion of ions in concrete conforms to Fick’s diffusion law, which is essentially the diffusion of ions under the action of concentration difference [46]. Therefore, the expression of ion diffusion depth *x*(*t*) can be obtained as follows:

(14) $$x\left(t\right)=2\sqrt{D\cdot t}\cdot erf^{-1}\left(1-\frac{c_{f}\left(x\left(t\right),t\right)-c_{0}}{c_{s}-c_{0}}\right)$$

where *x*(*t*) is the diffusion depth when the dry and wet cycle inspection period is *t* months (mm), *c_f_* (*x*(*t*),*t*) is the free ion content at the depth of *x*(*t*) when the dry and wet cycle inspection period is *t* months (%), *c_s_* is the surface ion content (%), *c*_0_ is the initial ion content (%), *D* is the ion diffusion coefficient (mm^2^/s), and *erf*^−1^(*u*) is the Gaussian error inverse function.

In Fick’s diffusion law [47], it is usually set that the free ion content on the concrete surface at the beginning is the initial free ion content in concrete *c*_0_, i.e., $c_{f}\left(x\left(0\right),0\right)=c_{0}$. The boundary condition is set as: at any time of *t*, the free ion content on the concrete surface is *c_s_*, i.e., $c_{f}\left(0,t\right)=c_{s}$; at any time of *t*, at an infinite depth, the content of free ions in concrete is *c*_0_, i.e., $c_{f}\left(\infty ,t\right)=c_{0}$, where *c*_0_ = 0 in this study. Hence, Equation (14) can be changed to:

(15) $$x\left(t\right)=2\sqrt{D\cdot t}\cdot erf^{-1}\left(1-\frac{c_{f}\left(x\left(t\right),t\right)}{c_{s}}\right)$$

**Figure 9:**
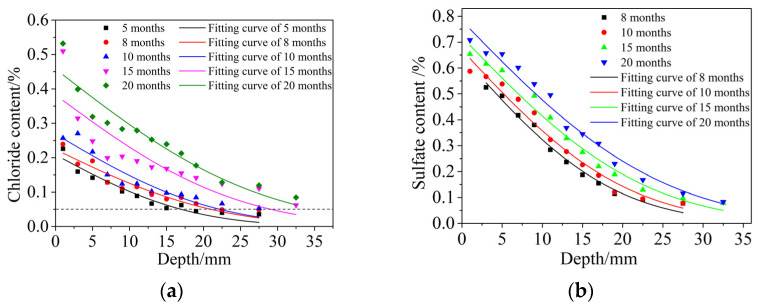
Distribution of free chloride and free sulfate contents versus the diffusion depth. (**a**) Free chloride, (**b**) Free sulfate.

The distribution of free chloride and free sulfate content versus the diffusion depth is shown in Figure 9. The content distribution law of free chloride and sulfate had the same regularity, and both conformed to Fick’s diffusion law. The free chloride and free sulfate content decreased gradually with increasing diffusion depths under the same dry and wet cycle inspection period, and the extent of decrease was smaller. At the same diffusion depth, with an increasing dry and wet cyclic corrosion duration, the contents of free chloride and sulfate gradually increased, and the increase range also increased. In this paper, it was assumed that all ions had the same diffusion depth after the same dry and wet cyclic corrosion duration, so chloride was selected for calculation. Fick’s second diffusion law was used to fit the distribution of chloride content in Figure 9a, and the regression values of surface chloride content *c_s_* and the diffusion coefficient *D* of chloride were obtained, as shown in Table 6.

Some scholars [48,49] studied the free chloride content at the discoloration interface through the AgNO_3_ color coloration method. The free chloride content here was defined as the damage depth. It was found that the free chloride content here accounts for 0.15~0.28% of the concrete quality. Therefore, $c_{f}\left(x\left(t\right),t\right)=0.15\%$ was also taken to define the damage depth *x_d_*(*t*).

Carlos [50] investigated actual concrete structures that had been corroded in different marine environments for longer than 50 years and found that the lowest concentration of chloride in all structures was 0.05%. Costa [51] obtained the corrosion depth of concrete when the concentration of chloride was 0.05%. Figure 9a shows that the horizontal dotted line, representing a 0.05% chloride concentration, intersected with the distribution curves of chloride contents in different dry and wet cyclic corrosion times at different points. The distribution curves of the chloride contents decreased very slowly and essentially did not change under the horizontal dotted line. Therefore, it is reasonable to take $c_{f}\left(x\left(t\right),t\right)=0.05\%$ as the corrosion depth *x_c_*(*t*).

When $c_{f}\left(x\left(t\right),t\right)=0.15\%$ (for damage depth) and 0.05% (for corrosion depth), and the surface chloride content *c_s_* and diffusion coefficient *D* of chloride in Table 6 were brought into formula (15), the damage depth *x_d_*(*t*) and corrosion depth *x_c_*(t) of concrete specimens at different dry and wet cyclic corrosion times could be obtained.

### 4.3. Results Verification and Analysis

**Figure 10:**
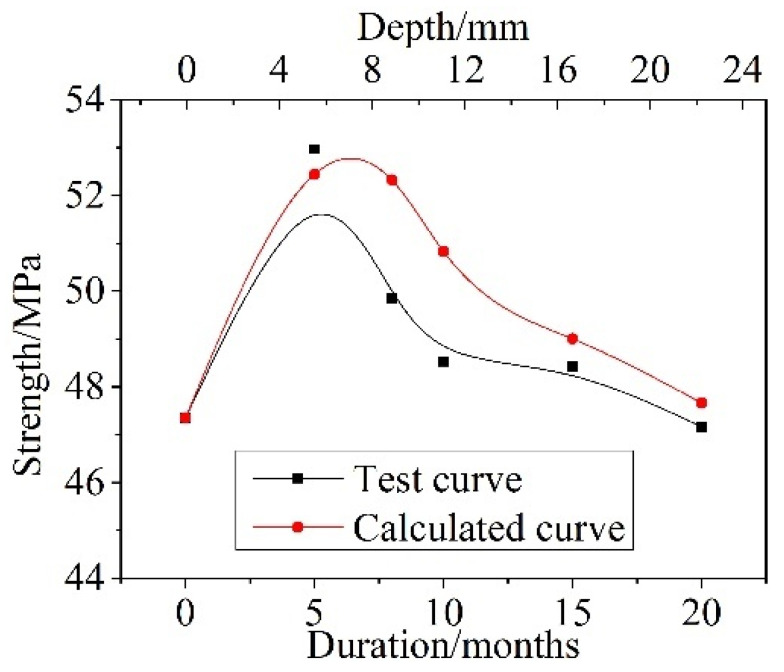
Comparison between the test values and calculated values of compressive strength after different dry and wet cycle inspection periods.

The compressive strength of concrete after different dry–wet cyclic corrosion durations can be calculated by introducing parameters of the damage depth *x_d_*(*t*), the corrosion depth *x_c_*(t), the corrosion damage coefficient *γ_d_*, and the corrosion enhancement coefficient *γ_c_* into Formula (10), and a comparison between the calculated and test values is shown in Figure 10. The calculated values were in good agreement with the test values, and both showed a trend of first increasing and then decreasing as the dry and wet cyclic corrosion duration increased. The maximum error was only approximately 5%.

## 5. Prediction and Analysis

### 5.1. Prediction of Damage Depth

#### 5.1.1. Time-Dependent Surface Chloride Content *c_s_*

**Figure 11:**
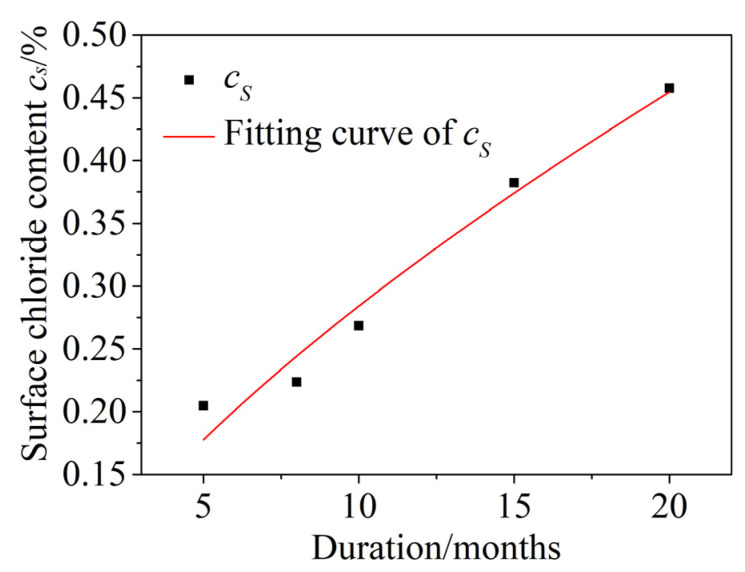
Time-dependent surface chloride content *c_s_.*

When concrete is exposed to an actual chloride environment, the surface chloride content *c_s_* is not constant, but is instead a time-dependent process from low to high and gradually to saturation. Costa [51] found that the relationship between the surface chloride concentration and corrosion exposure time was a power function, so a power function was used to fit the *c_s_* test data in this paper. The fitting curve is shown in Figure 11. The correlation coefficient is *R*^2^ = 0.958. The fitting function is given by Formula (16):

(16) $$c_{s}\left(t\right)=0.059t^{0.678}$$

#### 5.1.2. Time-Dependent Diffusion Coefficient D of Chloride

**Figure 12:**
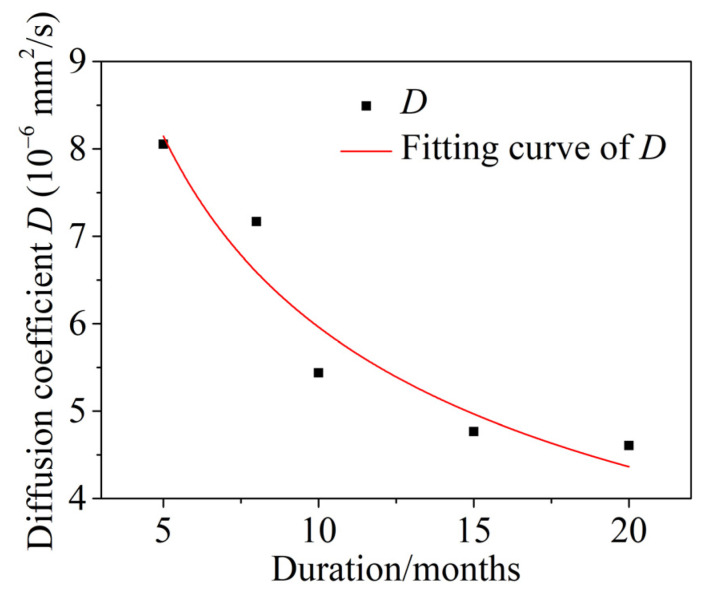
Time-dependent diffusion coefficient *D* of chloride.

The variation in the diffusion coefficient *D* of chloride after different dry and wet cyclic corrosion durations is shown in Figure 12. The results show that the diffusion coefficient *D* of chloride decreased gradually with increasing dry and wet cyclic corrosion durations and had obvious time dependence, i.e., the diffusion coefficient of chloride was not constant. In this paper, the diffusion coefficient *D* of chloride was modified by the attenuation coefficient *n* [52]. The fitting formulas are as follows:

(17) $$D_{t}=D_{{}^{0}}{\left(\frac{t_{0}}{t}\right)}^{n}=16.807\cdot {\left(\frac{1}{t}\right)}^{0.45}$$

where *D_t_* is the actual diffusion coefficient of chloride, *t*_0_ is the reference time (*t*_0_ = 1 in this paper), and *D*_0_ is the chloride diffusion coefficient with a dry and wet cycle inspection period of 1 month.

#### 5.1.3. Prediction of Damage Depth

The free chloride content for calculating the damage depth was given above, wherein $c_{f}\left(x\left(t\right),t\right)=0.15\%$. Then, the time-dependent formula of damage depth can be obtained by introducing Formulas (16) and (17) into Formula (15):

(18) $$x_{d}\left(t\right)=2\sqrt{16.807t^{0.55}}\cdot erf^{-1}\left(1-\frac{0.15}{0.059t^{0.678}}\right)$$

**Figure 13:**
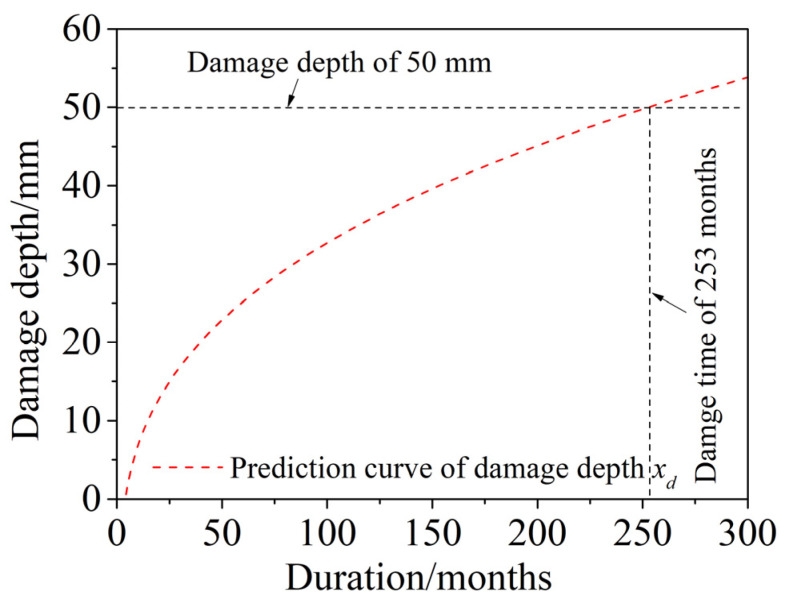
Prediction of time-dependent damage depth *x_d_.*

The prediction of the time-dependent damage depth *x_d_* is shown in Figure 13. The results show that, as the dry and wet cyclic corrosion duration increased, the damage depth increased gradually, with a slower rate, and gradually tended to stabilize. When *x*(*t*) = *a*/2, the corrosion damage zone extends to the whole specimen, and the specimen will crack completely. At this time, the dry and wet cyclic corrosion duration is approximately 253 months (21.1 years), which is much shorter than the 50 years of service life of normal concrete.

### 5.2. Prediction of Compressive Strength

The free chloride content for calculating the corrosion depth was given above, wherein $c_{f}\left(x\left(t\right),t\right)=0.05\%$. The time-dependent formula of corrosion depth can be obtained by referencing Formula (19):

(19) $$x_{c}\left(t\right)=2\sqrt{16.807t^{0.55}}\cdot erf^{-1}\left(1-\frac{0.05}{0.059t^{0.678}}\right)$$

The time-dependent formula of the compressive strength of corroded concrete in a saline soil area can be obtained by introducing Formulas (18) and (19) into Formula (10):

(20) $$\{\begin{array}{l} f_{cu}\left(t\right)=\left[1-0.1\cdot \frac{a^{2}-{\left(a-2x_{d}\left(t\right)\right)}^{2}}{a^{2}}+0.35\cdot \frac{{\left(a-2x_{d}\left(t\right)\right)}^{2}-{\left(a-2x_{c}\left(t\right)\right)}^{2}}{a^{2}}\right]\cdot f_{cu}\left(0\right)\\ x_{d}\left(t\right)=2\sqrt{16.807t^{0.55}}\cdot erf^{-1}\left(1-\frac{0.15}{0.059t^{0.678}}\right)\\ x_{c}\left(t\right)=2\sqrt{16.807t^{0.55}}\cdot erf^{-1}\left(1-\frac{0.05}{0.059t^{0.678}}\right) \end{array}$$

**Figure 14:**
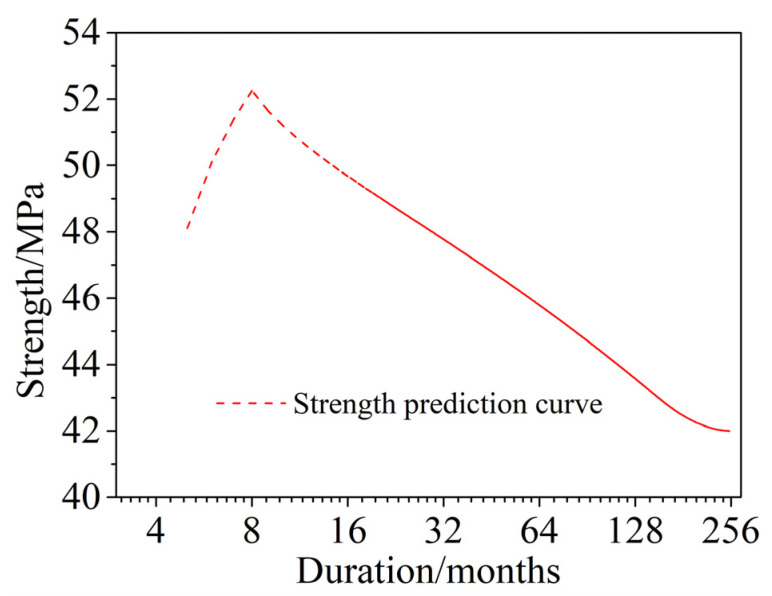
Prediction of time-dependent compressive strength of corroded concrete in a saline soil environment.

The prediction of the time-dependent compressive strength of corroded concrete in a saline soil environment is shown in Figure 14. The results show that, as the dry and wet cycle inspection period increases, the compressive strength increases rapidly at first, then decreases rapidly, and then decreases slowly. The rate of decline also becomes slower and tends to gradually stabilize. At approximately 187 months (15.6 years), the compressive strength essentially does not change, and the strength decreases to approximately 90% of the noncorroded concrete. Compared with the actual concrete compressive strength of Dun-huang Station [43] in the saline soil area of Western China, which was buried for 36 years, the deterioration time of concrete by the wet–dry cyclic corrosion test in this paper was obviously shorter, which was approximately 0.43 times the actual corrosion time of saline soil.

## 6. Conclusions

The variations in the concrete compressive strength versus the dry and wet cycle inspection periods in a saline soil environment was introduced, and a time-dependent compressive strength model of corroded concrete was established. The main conclusions are as follows:

1. The compressive strength of corroded concrete in a saline soil environment first increased and then decreased with increasing dry and wet cycle inspection periods. The deterioration of concrete in a saline soil environment developed gradually from the surface to the interior of the concrete. Therefore, the cross section of the concrete specimens was equivalent to three parts according to the damage depth and corrosion depth: a failure zone, a filling zone, and an undisturbed zone. The compressive strength of concrete was the superposition of these three parts.
2. The compressive strength of corroded concrete in a saline soil environment, as calculated by the mechanical model established in this paper, was in good agreement with the test results, showing a trend of first increasing and then decreasing as the dry and wet cyclic corrosion duration increased. The maximum error was less than five percent.
3. The service life of concrete was predicted to be 253 months (21.1 years) by damage depth and 187 months (15.6 years) by a time-dependent compressive strength model. Both predictions were far less than the normal concrete service life of 50 years. In addition, the long-term compressive strength of corroded concrete was found to be about 90% of that of noncorroded concrete, which did not deteriorate with the corrosion time.

## Figures and Tables

**Table 1:** Chemical composition of cement P. O. 42.5 R (%).

Chemical Composition	CaO	SiO_2_	Al_2_O_3_	Fe_2_O_3_	MgO	SO_3_	Loss on Ignition
Content	65.01	23.44	7.19	2.96	2.24	2.87	2.86

**Table 2:** Performance index of cement P. O. 42.5 R.

Density (kg/m^3^)	Specific Surface Area (m^2^/kg)	Setting Time (min)		Soundness	Compressive Strength (MPa)		Bending Strength (MPa)	
		Initial	Final		3 d	28 d	3 d	28 d
315.8	384	240	390	Fine	24.8	48.9	5.0	8.1

**Table 3:** Mix of the concrete (kg/m^3^).

Water	Cement	Fine Aggregate	Coarse Aggregate	Water/Cement Ratio
166	395	596	1263	0.42

**Table 4:** Mix of the test solution.

Salt Type (wt %)	NaCl	Na_2_SO_4_	MgCl_2_	MgSO_4_	NaHCO_3_	KCl	Total
Mass ratio	15	3	2	2	0.5	0.5	24

**Table 5:** Ion concentration in saline soil and the test solution.

Ion Concentration (g/L)	Na^+^	K^+^	Mg^2+^	Ca^2+^	Cl^−^	SO_4_^2−^	CO_3_^2−^	HCO_3_^−^	Total
Test solution	70.06	2.62	9.05	0	108.36	36.28	0.00	3.63	230
Saline soil	97.17	2.64	3.96	0.13	108.64	36.44	25.38	4.60	278.96

**Table 6:** Regression values of *D* and *c_s_.*

Inspection Periods *t* (Months)	5	8	10	15	20
*D* (10^−6^ mm/s)	8.0547	7.1679	5.4394	4.7668	4.6088
$c_{s}\;(\%)$	0.2084	0.2237	0.2686	0.3824	0.4577

## References

[1] Diab A.M., Elyamany H.E., Elmoaty A.E.M.A., Shalan A.H. (2014). Prediction of concrete compressive strength due to long term sulfate attack using neural network. Alex. Eng. J..

[2] Shazali M.A., Baluch M.H., Al-Gadhib A.H. (2006). Predicting Residual Strength in Unsaturated Concrete Exposed to Sulfate Attack. J. Mater. Civ. Eng..

[3] AAl-Shamiri b.K., Kim J.H., Yuan T., Yoon Y.S. (2019). Modeling the compressive strength of high-strength concrete: An extreme learning approach. Constr. Build. Mater..

[4] Pyo S., Tafesse M., Kim H., Kim H.-K. (2017). Effect of chloride content on mechanical properties of ultra high performance concrete. Cem. Concr. Compos..

[5] Lu C., Gao Y., Cui Z., Liu R. (2015). Experimental Analysis of Chloride Penetration into Concrete Subjected to Drying-Wetting Cycles. J. Mater. Civ. Eng..

[6] Müllauer W., Beddoe R.E., Heinz D. (2013). Sulfate attack expansion mechanisms. Cem. Concr. Res..

[7] Park Y.-S., Suh J.-K., Lee J.-H., Shin Y.-S. (1999). Strength deterioration of high strength concrete in sulfate environment. Cem. Concr. Res..

[8] Schmidt T., Lothenbach B., Romer M., Neuenschwander J., Scrivener K.L. (2009). Physical and microstructural aspects of sulfate attack on ordinary and limestone blended Portland cements. Cem. Concr. Res..

[9] Pruckner F., Gjørv O.E. (2004). Effect of CaCl_2_ and NaCl additions on concrete corrosivity. Cem. Concr. Res..

[10] Sanjuán M.A. (1997). Formation of chloroaluminates in calcium aluminate cements cured at high temperatures and exposed to chloride solutions. J. Mater. Sci..

[11] Neville A. (2014). The confused world of sulfate on concrete. Cem. Concr. Res..

[12] Flatt R.J. (2002). Salt damage in porous materials: How high supersaturations are generated. J. Cryst. Growth.

[13] Rodriguez-Navarro C., Doehne E., Sebastian E. (2000). How does sodium sulfate crystallize? Implications for the decay and testing of building materials. Cem. Concr. Res..

[14] Menéndez E., Argiz C., Sanjuán M.A., Menéndez E., Baroghel-Bouny V. (2020). Evolution of Damage Due to Sulphate Attack in Cement Mortar with and Without Ground Coal Bottom Ash. External Sulphate Attack-Field Aspects and Lab Tests.

[15] Al-Dulaijan S.U., Maslehuddin M., Al-Zahrani M.M., Sharif A.M., Shameem M., Ibrahim M. (2003). Sulfate resistance of plain and blended cements exposed to varying concentrations of sodium sulfate. Cem. Concr. Compos..

[16] Gollop R.S., Taylor H.F.W. (1992). Micro-structural and micro-analytical studies of sulfate attack I. Ordinary Portland cement paste. Cem. Concr. Res..

[17] Zajac M., Skocek J., Müller A., Haha M.B. (2018). Effect of sulfate content on the porosity distribution and resulting performance of composite cements. Constr. Build. Mater..

[18] Scherer G.W. (2004). Stress from crystallization of salt. Cem. Concr. Res..

[19] Flatt R.J., Scherer G.W. (2008). Thermodynamics of crystallization stresses in DEF. Cem. Concr. Res..

[20] Diab M.A., Awad A.E.M., Elyamany H.E., Elmoaty A.E.M.A. (2012). Guidelines in compressive strength assessment of concrete modified with silica fume due to magnesium sulfate attack. Constr. Build. Mater..

[21] Sotiriadis K., Nikolopoulou E., Tsivilis S. (2012). Sulfate resistance of limestone cement concrete exposed to combined chloride and sulfate environment at low temperature. Cem. Concr. Compos..

[22] Li Y., Guan Z., Wang Z., Wang P., Li Y., Zhang G., Ding Q. (2019). 3D meso-scale finite element modelling on cement paste corroded in sodium sulfate with X-ray CT technique. Constr. Build. Mater..

[23] Zhao G., Li J., Han F., Shi M., Fan H. (2019). Sulfate-induced degradation of cast-in-situ concrete influenced by magnesium. Constr. Build. Mater..

[24] Yu X.-t., Chen D., Feng J.-r., Zhang Y., Liao Y.-d. (2018). Behavior of mortar exposed to different exposure conditions of sulfate attack. Ocean Eng..

[25] Xie F., Li J., Li L., Zhao G., Yao M. (2019). Numerical solution and damage evaluation for cast-in-situ piles exposed to external sulfate attack. Constr. Build. Mater..

[26] Wang D., Zhou X., Meng Y., Chen Z. (2017). Durability of concrete containing fly ash and silica fume against combined freezing-thawing and sulfate attack. Constr. Build. Mater..

[27] Zhang Z., Jin X., Luo W. (2019). Long-term behaviors of concrete under low-concentration sulfate attack subjected to natural variation of environmental climate conditions. Cem. Concr. Res..

[28] Ikumi T., Segura I. (2019). Numerical assessment of external sulfate attack in concrete structures. A review. Cem. Concr. Res..

[29] Ma H., Gong W., Yu H., Sun W. (2018). Durability of concrete subjected to dry-wet cycles in various types of salt lake brines. Constr. Build. Mater..

[30] Liu L.-x. (2001). Brief introduction on the study of erosion and prevention of concrete in salt lake and saline soil area of chaerhan, chaidamu. J. Build. Mater..

[31] Liu Z., Zhang F., Deng D., Xie Y., Long G. (2017). Physical sulfate attack on concrete lining-a field case analysis. Case Stud. Constr. Mater..

[32] Zhang L., Su X. (2014). Comparison and selection of the durability evaluating parameters of concrete in saline soil region. Adv. Mater. Res..

[33] Jin W.F., Wang Y.H., Zhou G., Fan K. (2012). Experimental study on anti-frozen durability of concrete in saline soil region. Adv. Mater. Res..

[34] Yang D., Yan C., Liu S., Zhang J., Hu Z. (2019). Stress-strain constitutive model of concrete corroded by saline soil under uniaxial compression. Constr. Build. Mater..

[35] (2007). Common Portland Cement.

[36] Hongfa Y.U. (2004). Study on High Performance Concrete in Salt Lake: Durability, Mechanism and Service Life Prediction. Ph.D. Thesis.

[37] (2006). Test Code for Hydraulic Concrete.

[38] Otsuki N., Nagataki S., Nakashita K. (1993). Evaluation of AgNO_3_ solution spray method for measurement of chloride penetration into hardened cementitious matrix materials. Constr. Build. Mater..

[39] (2016). Standard for Test Method of Mechanical Properties on Ordinary Concrete.

[40] Sanjuán M.A. (2000). Electrochemical method to assess the absorption of NaCl solutions in OPC and SRPC mortars. Build. Environ..

[41] Ikumi T., Cavalaro S.H.P., Segura I. (2019). The role of porosity in external sulphate attack. Cem. Concr. Compos..

[42] Andrade C., Sagrera J.L., Sanjuán M.A., Andrade C., Kropp J. (2000). Several years study on chloride ion penetration into concrete exposed to Atlantic Ocean water. Proceedings of the PRO 19 2nd International RILEM Workshop on Testing and Modelling the Chloride Ingress into Concrete, Paris, France, 11–12 September 2000.

[43] Ma X., Qiu X., Chen C. (1998). Date accumulation and research on corrosion regularity for concrete and reinforced concrete in soil. Build. Sci..

[44] Kwon S.-J., Lee H.-S., Karthick S., Saraswathy V., Yang H.-M. (2017). Long-term corrosion performance of blended cement concrete in the marine environment—A real-time study. Constr. Build. Mater..

[45] Kumar R., Bhattacharjee B. (2003). Porosity, pore size distribution and in situ strength of concrete. Cem. Concr. Compos..

[46] Andrade C., Sanjuán M.A., Recuero A., Río O. (1994). Calculation of chloride diffusivity in concrete from migration experiments, in non steady-state conditions. Cem. Concr. Res..

[47] Poulsen E., Mejlbro L. (2010). Diffusion of Chloride in Concrete: Theory and Application.

[48] Sirivivantnanon V., Khatri R. Chloride penetration resistance of concrete. Proceedings of the Concrete Institute of Australia Conference ‘Getting a Lifetime out of Concrete Structures’.

[49] Andrade C., Castellote M., Alonso C., González C. (1999). Relation between colorimetric chloride penetration depth and charge passed in migration tests of the type of standard ASTM C1202–91. Cem. Concr. Res..

[50] Balestra C.E.T., Reichert T.A., Savaris G. (2019). Contribution for durability studies based on chloride profiles analysis of real marine structures in different marine aggressive zones. Constr. Build. Mater..

[51] Costa A., Appleton J. (1999). Chloride penetration into concrete in marine environment—Part II: Prediction of long term chloride penetration. Mater. Struct..

[52] Luping T., Gulikers J. (2007). On the mathematics of time-dependent apparent chloride diffusion coefficient in concrete. Cem. Concr. Res..

